# SGLT-2i and Risk of Malignancy in Type 2 Diabetes: A Meta-Analysis of Randomized Controlled Trials

**DOI:** 10.3389/fpubh.2021.668368

**Published:** 2021-06-07

**Authors:** Nanjing Shi, Yetan Shi, Jingsi Xu, Yuexiu Si, Tong Yang, Mengting Zhang, Derry Minyao Ng, Xiangyuan Li, Fei Xie

**Affiliations:** ^1^Department of Endocrinology, Affiliated Hangzhou First People' Hospital, Zhejiang University School of Medicine, Hangzhou, China; ^2^The Second Clinical Medical College, Zhejiang Chinese Medical University, Hangzhou, China; ^3^School of Basic Medical Sciences, Zhejiang Chinese Medical University, Hangzhou, China; ^4^Department of Tumor High Intensity Focused Ultrasound Therapy, HwaMei Hospital, University of Chinese Academy of Sciences, Ningbo, China; ^5^Medical College of Ningbo University, Ningbo, China; ^6^Department of Endocrinology, Ningbo Yinzhou No. 2 Hospital, Ningbo, China

**Keywords:** SGLT-2i, type 2 diabetes, malignant tumor, meta-analysis, RCT

## Abstract

**Background:** Currently, the association between sodium-glucose cotransporter 2 inhibitor (SGLT-2i) and malignancy risk has yet to be fully elucidated. This meta-analysis aimed to determine the relationship between SGLT-2i and malignancy risk in type 2 diabetes (T2D) patients.

**Methods:** We searched PubMed, ScienceDirect, EMBASE, Cochrane Central Register of Controlled Trials, and Web of Science to identify randomized controlled trials (RCTs) published up to August 2020 related to T2D patients treated with SGLT-2i vs. placebo or other hypoglycemic agents. The meta-analysis's primary outcome was malignancies' incidence, and the results were evaluated using risk ratio (RR) and 95% confidence interval (CI).

**Results:** We reviewed 76 articles (77 RCTs), comprising 45,162 and 43,811 patients in SGLT-2i and control groups, respectively. Compared with the control group, SGLT-2i had no significant association with augmented overall malignancy risk in T2D patients (RR = 1.05, 95% CI = 0.97–1.14, *P* = 0.20), but ertugliflozin may upsurge the risk (RR = 1.80, 95% CI = 1.02–3.17, *P* = 0.04). Compared with active hypoglycemic agents, dapagliflozin may increase (RR = 2.71, 95% CI = 1.46–6.43, *P* = 0.02) and empagliflozin may decrease (RR = 0.67, 95% CI = 0.45–0.98, *P* = 0.04) the malignancy risk. Compared with placebo, empagliflozin may exhibit risk increase (RR = 1.25, 95% CI = 1.05–1.49, *P* = 0.01), primarily in digestive system (RR = 1.48, 95% CI = 0.99–2.21, *P* = 0.05).

**Conclusions:** Our results proposed that in diverse comparisons, ertugliflozin and dapagliflozin seemed to increase the malignancy risk in T2D patients. Empagliflozin may cause malignancy risk reduction compared with active hypoglycemic agents but increase overall risk primarily in the digestive system compared with placebo. In short, the relationship between SGLT-2i and malignancy in T2D patients remains unclear.

## Introduction

The incidence of diabetes rises annually, with about 463 million people living with the disease today and an estimated 578 million by 2030 (1). Poor blood sugar control in diabetics may cause blindness, kidney failure and lower limb amputations (2). In recent decades, type 2 diabetes (T2D) has become a global public health crisis with a severe impact on human health (3), accounting for about 90% of people with diabetes, and the second leading global death reason is cancer, representing one sixth (4). Diabetes is evidenced to associate with a potential malignancy, risk with diabetics having a 10–20% higher risk of malignancy than non-diabetics (5). Studies have demonstrated that T2D significantly increases specific cancers' risk, such as liver and pancreatic cancer (6). The tumor-causing mechanisms of T2D may include hyperinsulinemia, insulin resistance, hyperglycemia, oxidative stress, and chronic inflammation (7).

Sodium-glucose cotransporter 2 inhibitor (SGLT-2i) can selectively inhibit glucose renal reabsorption and increase urine glucose excretion, independent of insulin action to reduce the blood sugar level of drug (8). In addition to reducing blood sugar and weight and lowering blood pressure, studies have revealed that SGLT-2i is beneficial in slowing the progression of cardiovascular and kidney diseases (9, 10). Based on the above advantages, SGLT-2i has a great application prospect. Evidence proposes that SGLT-2i is not significantly associated with increased overall cancer risk (11). However, some SGLT-2i can increase or decrease certain cancers' risk, such as dapagliflozin, which may increase the risk of bladder cancer and breast cancer in T2D patients (12), and canagliflozin may reduce the risk of gastrointestinal cancers (11). Given the low incidence of malignant tumors and the long incubation period, a longer follow-up time is mandatory. Our meta-analysis was conducted to investigate SGLT-2i impact on malignancy incidence in T2D patients.

## Methods

### Search Strategy

This meta-analysis was reported according to the Preferred Reporting Items for Systematic Reviews and Meta-Analyses guidelines (13). The included randomized controlled trials (RCTs) were SGLT-2i in T2D patients. SGLT-2i such as dapagliflozin, canagliflozin, empagliflozin, tofogliflozin, ertugliflozin, luseogliflozin, and bexagliflozin were compared with placebo or other active hypoglycemic drugs to explore malignancy incidence in patients during follow-up.

After conducting a comprehensive and systematic search in PubMed, ScienceDirect, EMBASE, Cochrane Central Register of Controlled Trials and Web of Science databases, only articles published in English by August 2020 and before are retrieved. The search formula was as follows: (type 2 diabetes OR type 2 diabetes mellitus OR T2DM OR T2D) AND (sodium-glucose cotransporter 2 inhibitor OR SGLT-2i OR sotagliflozin OR janagliflozin OR dapagliflozin OR canagliflozin OR empagliflozin OR ipragliflozin OR tofogliflozin OR ertugliflozin OR luseogliflozin OR sergliflozin OR licogliflozin OR remogliflozin OR bexagliflozin). Two researchers independently searched the articles, reviewed the title and abstract, viewed the full text, and selected the inclusion articles. To avoid missing negative results, the vocabulary related to malignant tumors was not limited. Instead, the full text (including Supplementary Materials) was scanned to extract relevant data.

### Study Selection

Studies that fulfill the following criteria were encompassed in this meta-analysis: (1) participants were T2D patients; (2) RCTs compared the therapeutic efficacy of SGLT-2i with placebo or other hypoglycemic agents; (3) RCTs stated thorough information on malignancy occurrence; (4) the experimental group was provided SGLT-2i therapy (including single drug or combination drug), and the control group was supplied non-SGLT-2i therapy (placebo or other hypoglycemic drugs). Exclusion criteria: (1) non-RCTs, including review, observational research, cases; (2) patients with type 1 diabetes mellitus or healthy volunteers; (3) non-English language; (4) duplicate reports. When articles were repeatedly updated, the most recent or data-complete one was involved herein. After a systematic search, the two authors evaluated all chosen works, and the questionable studies were further discussed to resolve various opinions.

### Data Extraction and Quality Assessment

Data extraction for studies included was performed independently by two researchers and reviewed by a third one. The extracted data comprise (1) study characteristics, such as author, region, year of publication, and follow-up time; (2) participant characteristics, including age, gender and subject inclusion criteria; (3) total number of malignant neoplasms, including primary, recurrent and metastatic cancers and classification of different types of tumors; (4) drug dose utilized by the experimental and control groups.

RCTs were assessed utilizing the Cochrane Collaboration's tool. The evaluation criteria include review and judgment of “low risk,” “high risk,” or “unclear risk” in terms of sequence generation, allocation concealment, blinding, incomplete outcome data, as well as selective outcome reporting and free of other bias. Any differences between the two researchers were resolved by discussion or by a third person review.

### Statistical Analysis

The Review Manager 5.3 statistical analysis software was employed for the above analysis. The risk ratio (RR) and 95% confidence interval (CI) were deployed to evaluate the results. Heterogeneity of included studies was assessed employing *I*^2^ statistics, where *I*^2^ < 50%, indicating low heterogeneity, and a fixed-effect model was used; otherwise, a random-effect model was utilized. *P* ≤ 0.05 was statistically significant, and *P* < 0.10 within the suspected influence scope.

## Results

### Eligible Studies and Characteristics

**Figure 1:**
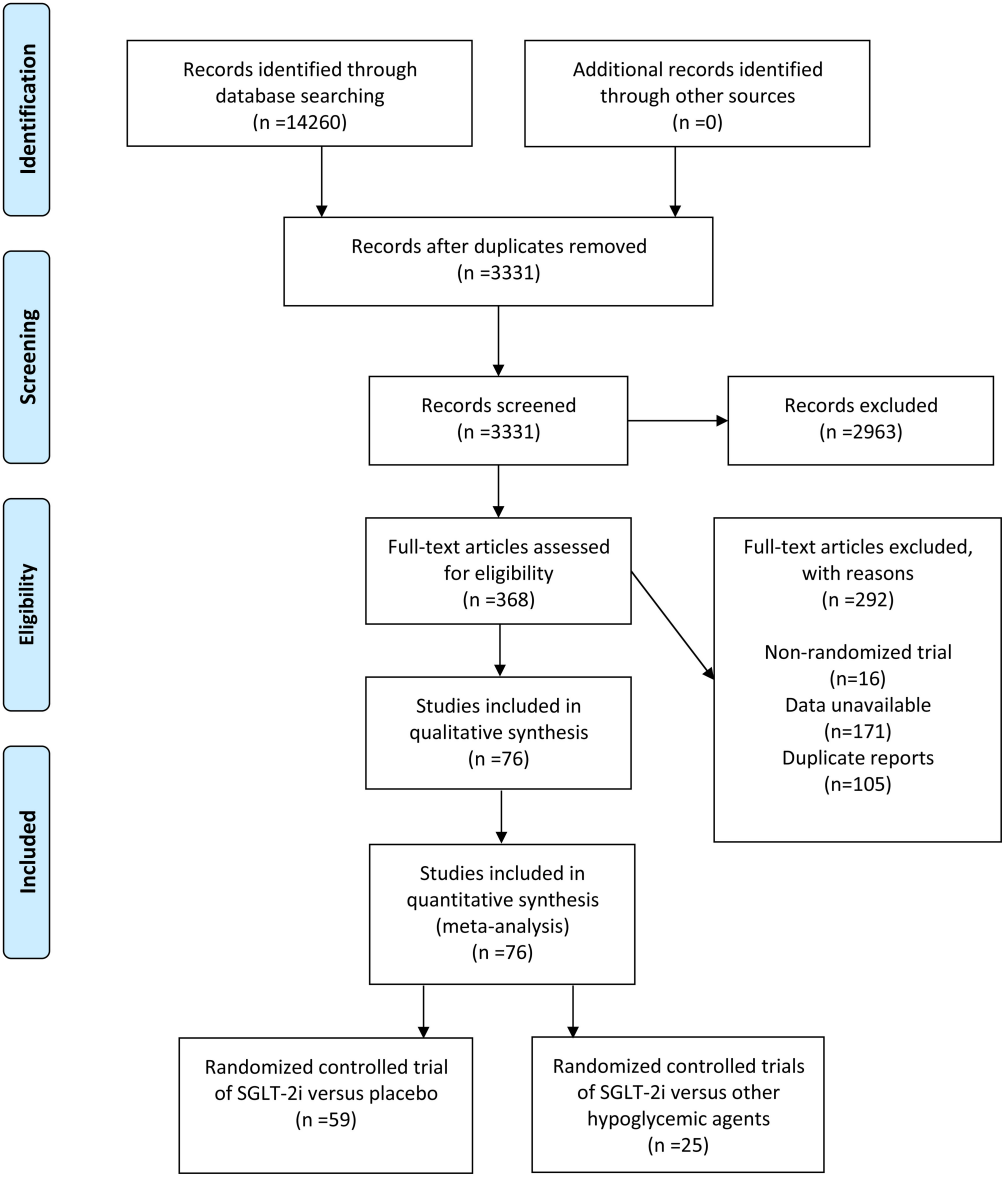
A schematic flow for selecting the articles included in this meta-analysis.

A total of 14,260 articles were initially searched, leaving 3,331 articles after deletion of duplicates. By reviewing title and abstract information, 2,963 articles were excluded. By evaluating 368 full-text articles, 292 articles were excluded, comprising 16 non-RCTs, 171 articles provided data without malignant tumors, and 105 repeated reports. Finally, 76 articles between 2012 and 2020 were chosen for this meta-analysis (14–89), with 77 RCTs. Typically, 59 articles existed on SGLT-2i vs. placebo [60 RCTs in total, one article containing 2 RCTs (64)], and 25 articles existed on SGLT-2i vs. other hypoglycemic agents (Figure 1).

A total of 88,973 participants were included in 77 trials, of which 45,162 were randomly assigned to an intervention group comprising SGLT-2i, such as ertugliflozin, bexagliflozin, dapagliflozin, empagliflozin, canagliflozin, tofogliflozin, and luseogliflozin. A total of 43,811 patients were randomly assigned to a control group, including glucagon-like peptide-1 receptor agonist (GLP-1RA), dipeptidyl-peptidase-4 inhibitor (DPP-4i), sulfonylurea, thiazolidinedione, metformin, or placebo (part of the study was drug combination therapy). The RCTs were conducted in different countries, including Japan (13 RCTs), the United Kingdom (2 RCTs), and the United States (1 RCT). The remaining 61 were multi-national multicenter studies. Follow-up time ranged from 10 to 416 weeks, with 30 short-term studies (<52 weeks), 31 mid-term studies (52–104 weeks), and 16 long-term studies (≥104 weeks) (Table 1).

**Table 1:** Characteristics of all the studies included in the meta-analysis.

**Author**	**Country**	**ClinicalTrials.gov identifier**	**Inclusion criteria**	**Follow-up time (week)**	**Therapeutic regimen**	
					**Experiment**	**Control**
Allegretti et al. (14)	Multicenter	NCT02836873	eGFR 30–59 ml/min/1.73 m^2^; no change treatment (≥8 weeks)	26	Bexagliflozin 20 mg	Placebo
Araki et al. (15)	Japan	NCT01368081	HbA1c 7.0–10.0%; diet and exercise and monotherapy with an SU, biguanide, TZD, AGI, DPP-4i, or glinide; no change background antidiabetes therapies for 10 weeks; receive antihypertensive therapy for 4 weeks before randomization	53	Empagliflozin 10 mg/25 mg (SU)	MET (SU)
					Empagliflozin 10 mg/25 mg (Biguanide)	
					Empagliflozin 10 mg/25 mg (TZD)	
					Empagliflozin 10 mg/25 mg (AGI)	
					Empagliflozin 10 mg/25 mg (DPP-4i)	
					Empagliflozin 10 mg/25 mg (Glinide)	
Aronson et al. (16)	Multicenter	NCT01958671	HbA1c 7.0–10.5%; not use OAD (≥8 weeks) or use OAD only once	54	Ertugliflozin 5 mg/15 mg	Placebo/MET
Bailey et al. (17)	Multicenter	NCT00528879	MET ≥1,500 mg/day (≥8 weeks); C-peptide ≥1.0 ng/ml; Scr <1.50 mg/dl for men or <1.40 mg/dl for women	102	Dapagliflozin 2.5 mg/5 mg/10 mg + MET	Placebo + MET
Bailey et al. (18)	Multicenter	NCT00528372	peptide ≥1.0 ng/ml; drug naive; Group 1: HbA1c ≥7% and ≤ 10%; Group 2: HbA1c ≥10.1% and ≤ 12.0%	106	Group 1: Dapagliflozin 2.5 mg/5 mg/10 mg AM	Group 1: Placebo AM & PM
					Group 1: Dapagliflozin 2.5 mg/5 mg/10 mg PM	
					Group 2: Dapagliflozin 5 mg/10 mg AM	
Barnett et al. (19)	Multicenter	NCT01164501	HbA1c 7.0–10.0%; eGFR <90 ml/min; diet and exercise; pre-treated with any antidiabetic therapy and no change for 12 weeks	52	Empagliflozin 10 mg/25 mg	Placebo
Bolinder et al. (20)	Multicenter	NCT00855166	HbA1c 6.5–8.5%; women 55–75 years (post-menopausal ≥5 years); men 30–75 years; FPG ≤ 13.2 mmol/l; body weight ≤ 120 kg; treatment with MET ≥1,500 mg/day (≥12 weeks)	102	Dapagliflozin 10 mg + MET	Placebo + MET
Brown et al. (21)	UK	NCT02956811	HbA1c 6.5–10.0%; BP <145/90 mmHg; echocardiographic LV hypertrophy	52	Dapagliflozin 10 mg	Placebo
Böhm et al. (22)	Multicenter	NCT01131676	CVD; eGFR of at least 30 ml/min/1.73 m^2^	260	Empagliflozin 10 mg/25 mg	Placebo
Cahn et al. (23)	Multicenter	NCT01730534	high CV risk; HbA1c 6.5–12.0%; creatinine clearance rate 60 ml/min	270	Dapagliflozin 10 mg	Placebo
Cefalu et al. (24)	Multicenter	NCT01031680	cerebrovascular disease; hypertension	52	Dapagliflozin 10 mg	Placebo
Dagogo-Jack et al. (25)	Multicenter	NCT02036515	HbA1c 7.0–10.5%; MET 1,500 mg/day and sitagliptin 100 mg/day for 8 weeks	54	Ertugliflozin 5 mg/15 mg	Placebo
Ferdinand et al. (26)	US	NCT02182830	HbA1c 7.0–11.0%; hypertension; SBP 140–180 mmHg	25	Empagliflozin 10–25 mg	Placebo
Ferrannini et al. (27)	Multicenter	NCT00881530	HbA1c 7.0–10.0%; drug naive or MET ≥1,500 mg/day or maximum tolerated dose ≥10 weeks	79	Empagliflozin 10 mg/25 mg	MET
					Empagliflozin 10 mg/25 mg + MET	Sitagliptin 100 mg + MET
Fioretto et al. (28)	Multicenter	NCT02413398	HbA1c 7.0–11%; stable antidiabetic treatment; renal impairment: CKD 3A	28	Dapagliflozin 10 mg	Placebo
Forst et al. (29)	Multicenter	NCT01106690	HbA1c 7–10.5%; pioglitazone or rosiglitazone and another AHA (MET); FPG <15 mmol/l	52	Canagliflozin 100 mg/300 mg	Placebo/Sitagliptin 100 mg
Fuchigami et al. (30)	Japan	NA	HbA1c 7.1–10.0%; not use any AHA within 8 weeks or only use MET	24	Dapaglifozin 5–10 mg	Sitagliptin 50–100 mg
Gallo et al. (31)	Multicenter	NCT02033889	HbA1c 7.0–10.5%; MET (<8 weeks) or change diabetes regimen	106	Ertugliflozin 5 mg/15 mg	Placebo/Glimepiride
Grunberger et al. (32)	Multicenter	NCT01986855	CKD 3; eGFR 30–60 ml/min/1.73 m^2^; HbA1c 7.0–10.5%; diet and exercise or with AHA monotherapy or combination therapy using other AHAs (INS and SU)	54	Ertugliflozin 5 mg/15 mg	Placebo
Hadjadj et al. (33)	Multicenter	NCT01719003	HbA1c 7.5–12%; diet and exercise; drug-naive	25	Empagliflozin 12.5 mg/5 mg BID + MET 1,000 mg BID	MET 1,000 mg BID
					Empagliflozin 12.5 mg/5 mg BID + MET 500 mg BID	MET 500 mg BID
					Empagliflozin 10 mg/25 mg QD	
Halvorsen et al. (34)	Multicenter	NCT01377844	HbA1c 7–10%; not treat with OAD: FPG <13.9 mmol/l; treat with OAD: FPG <13.3 mmol/l; antidiabetic or antihypertensive or antihyperlipidemic regimen must be stable (≥3 month);capillary blood glucose <13.9 mmol/l	96	Bexagliflozin 20 mg	Placebo
Halvorsen et al. (35)	Multicenter	NCT03115112	MET ≥1,500 mg/day no change at 8 weeks; hypertension or hyperlipidemia medications must be stable (≥1 month) (if applicable)	24	Bexagliflozin 20 mg	Sitagliptin 100 mg
Halvorsen et al. (36)	Multicenter	NCT02390050	naive or take one OAD in combination with diet and exercise; naive: HbA1c 7.0–8.5%; one OAD: HbA1c 6.5–8.5%; hypertension or hyperlipidemia medications must be stable (≥1 month)	14	Bexagliflozin 5 mg/10 mg/20 mg	Placebo
Haneda et al. (37)	Japan	NA	HbA1c 6.5–10.0%; eGFR 30–60 ml/min/1.73 m^2^; diet and exercise only or treat with 1 or 2 OHAs at a fixed dose >8 weeks	52	Luseogliflozin 2.5 mg	Placebo/Luseogliflozin
Henry et al. (38)	Multicenter	NCT00643851	drug naive or with AHA for <24 weeks; C-peptide ≥1.0 ng/ml; Scr <1.50 mg/dl for men or <1.40 mg/dl for women	28	Dapagliflozin 5 mg + MET XR	MET XR
					Dapagliflozin 5 mg	
Hollander et al. (39)	Multicenter	NCT01999218	HbA1c 7.0–9.0%; MET monotherapy 1,500 mg/day for 8 weeks or with an AHA	106	Ertugliflozin 5 mg/15 mg	Glimepiride
Ikeda et al. (40)	Multicenter	NCT00800176	HbA1c 7.0–10.0%; diet and exercise or with stable MET (≥3 month)	12	Tofogliflozin 2.5 mg/5 mg/10 mg/20 mg/40 mg	Placebo
Inagaki et al. (41)	Japan	NCT01022112	HbA1c 6.9–9.9%; diet and exercise; no change regimen for ≥8 weeks	14	Canagliflozin 50 mg/100 mg/200 mg/300 mg	Placebo
Inagaki et al. (42)	Japan	NCT01413204	HbA1c 7.0–10.0%; diet and exercise for 55 days	26	Canagliflozin 100 mg/200 mg	Placebo
Jabbour et al. (43)	Multicenter	NCT00984867	Not receive treatment, or receive MET, sitagliptin or vildagliptin or the combination of these; blood test: need additional therapy	48	Dapagliflozin 10 mg	Placebo
Jabbour et al. (44)	Multicenter	NCT02229396	HbA1c 8.0–12.0%; MET ≥1,500 mg/day (≥2 months)	104	Dapagliflozin 10 mg + Placebo	Exenatide 2 mg + Placebo
					Dapagliflozin 10 mg + Exenatide 2 mg	
Januzzi et al. (45)	Multicenter	NCT01106651	HbA1c 7–10.0%; no AHA or on a stable regimen of monotherapy or combination therapy; FPG <15 mmol/l; eGFR ≥50 ml/min/1.73 m^2^	104	Canagliflozin 100 mg/300 mg	Placebo
Ji et al. (46)	Multicenter	NCT01095653	HbA1c 7.5–10.5%; C-peptide level ≥1.0 ng/ml; drug naive	28	Dapagliflozin 5 mg/10 mg	Placebo
Ji et al. (47)	Multicenter	NCT02630706	MET (≥1,500 mg/day): HbA1c 7.0–10.5%; MET <1,500 mg/day: HbA1c 7.5–11.0%; dual combination therapy with MET + SU, DDP-4i, meglitinide, or AGI: HbA1c 6.5–9.5%	28	Ertugliflozin 5 mg/15 mg	Placebo
Kadowaki et al. (48)	Japan	NCT01193218	diet and exercise; drug naive HbA1c 7.0–10.0%; one AHA: HbA1c 6.5–9.0%; Visit 2: HbA1c 7.0–10%	52	Empagliflozin 5 mg/10 mg/25 mg/50 mg	Placebo
Kadowaki et al. (49)	Japan	NCT02354235	HbA1c 7.0–10.5%; FPG ≤ 15 mmol/l; diet and exercise; teneligliptin 20 mg monotherapy once daily (≥8 weeks)	26	Canagliflozin 100 mg + Teneligliptin 20 mg	Placebo + Teneligliptin 20mg
Kaku et al. (50)	Japan	NCT00972244	strictly/relatively treatment naive: HbA1c 7.0–10%; with single or two AHA: HbA1c ≤ 8%; FPG ≤ 13.3 mmol/l; C-peptide >1.0 ng/ml; Scr <1.5 mg/dl for men and <1.4 mg/dl for women; eGFR >60 ml/min/1.73 m^2^	16	Dapagliflozin 1 mg/2.5 mg/ 5 mg/10 mg	Placebo
Kaku et al. (51)	Japan	NA	HbA1c 7.3–10.3%; diet and exercise only ≥8 weeks; percent change: HbA1c ≤ 10% and body weight <5% from the provisional registration visit to the final registration visit; no changes in antihypertensive medications; stop other AHAs ≥8 weeks	26	Tofogliflozin 10 mg/20 mg/40 mg	Placebo
Katakami et al. (52)	Japan	NA	HbA1c 6–9% with diet and exercise without being on drugs or on SGLT-2i in the past but without them ≥12 weeks; no change in the antidiabetic, antithrombotic, antihypertensive, anti-dyslipidemia medication ≥12 weeks	104	Tofoglifozin 20 mg	Conventional
Kawamori et al. (53)	Japan	NCT02453555	diet and exercise and either treatment-naive or use one OAD for ≥12 weeks; treatment-naive: HbA1c 8.0–10.5%; OAD-pretreated (except linagliptin): HbA1c 7.5–10.5%; linagliptin-pretreated: HbA1c 7.5–10.0%	53	Empagliflozin 10 mg/25 mg + Linagliptin 5 mg	Placebo + Linagliptin 5 mg
Kohan et al. (54)	Multicenter	NCT00663260	HbA1c 7.0–11.0%; eGFR 30–59 ml/min/1.73 m^2^; diet and exercise or with a regimen of any approved AHAs, no change for 6 weeks	104	Dapagliflozin 5 mg/10 mg	Placebo
Lavalle-González et al. (55)	Multicenter	NCT01106677	HbA1c 7–10.5%; MET therapy ≥2,000 mg/day or ≥1,500 mg/day for ≥8 weeks; FPG <15 mmol/l at week −2 and fasting fingerstick glucose ≥6.1 mmol/l and <15 mmol/l on day 1	52	Canagliflozin 100 mg	Placebo/Sitagliptin 100 mg
					Canagliflozin 300 mg	Sitagliptin 100 mg
Leiter et al. (56)	Multicenter	NCT01042977	CVD; antidiabetic treatment (≥8 weeks); HbA1c 7.0–10.0%	52	Dapagliflozin 10 mg	Placebo
Leiter et al. (57)	Multicenter	NCT00968812	MET ≥2,000 mg/day or ≥1,500 mg/day for ≥10 weeks; HbA1c 7.0–9.5%; FPG ≤ 15 mmol/l at week −2	104	Canagliflozin 100 mg/300 mg	Glimepiride
Lewin et al. (58)	Multicenter	NCT01422876	HbA1c 7.0–10.5%; diet and exercise with drug-naive or pre-treated with MET unchange for 12 weeks	53	Empagliflozin 10 mg/25 mg + Linagliptin 5 mg (MET)	Linagliptin 5 mg (MET)
					Empagliflozin 10 mg/25 mg (MET)	
					Empagliflozin 10 mg/25 mg +Linagliptin 5 mg (Treatment Naive)	Linagliptin 5 mg (Treatment Naive)
					Empagliflozin 10 mg/25 mg (Treatment Naive)	
Lingvay et al. (59)	Multicenter	NCT03136484	HbA1c 7.0–10.5%; MET ≥1,500 mg/day or maximum tolerated dose for ≥90 days; eGFR ≥60 ml/min/1.73 m^2^	57	Canagliflozin 300 mg	Semaglutide 1 mg
Mathieu et al. (60)	Multicenter	NCT01646320	Stratum A: HbA1c 8.0–11.5%, MET ≥1,500 mg/day therapy alone (≥8 weeks) Stratum B: HbA1c 7.5–10.5%; MET ≥1,500 mg/day therapy and a DPP-4i (≥8 weeks); C-peptide ≥1.0 ng/ml	52	Dapagliflozin 10 mg + Saxagliptin 5 mg+ MET ≥1,500 mg	Placebo + Saxagliptin 5 mg + MET ≥1,500 mg
Matthaei et al. (61)	Multicenter	NCT01392677	HbA1c 7.0–10.5%; MET ≥1,500 mg/day and a maximum tolerated dose of SU (≥8 weeks)	52	Dapagliflozin 10 mg + MET + SU	Placebo + MET + SU
Müller-Wieland et al. (62)	Multicenter	NCT02471404	HbA1c 7.5–10.5%; MET ≥1,500 mg/day (≥8 weeks); C-peptide ≥1.0 ng/ml; FPG ≤ 15 mmol/l	52	Dapagliflozin 10 mg	Glimepiride 1 mg/2 mg/4 mg
					Saxagliptin 5 mg + Dapagliflozin 10 mg	
Nauck et al. (63)	Multicenter	NCT00660907	HbA1c 6.5–10%; FPG ≤ 15 mmol/l; C-peptide ≥1.0 ng/ml; MET or MET plus one other OAD, administer up to half-maximal dose (≥8 weeks)	208	Dapagliflozin 2.5 mg/5 mg/ 10 mg + MET	Glipizide 5 mg/10 mg/20 mg+ MET
Oshima et al. (64)	Multicenter	NCT01032629	HbA1c 7.0–10.5%; ≥30 years with history of CV event, or ≥50 years old with high risk of CV events; not on diabetes drug therapy or on therapy with any approved class of diabetes drugs	416	Canagliflozin 100 mg/300 mg	Placebo
Oshima et al. (64)	Multicenter	NCT01989754	HbA1c 7.0–10.5%; ≥30 years with history of CV event, or ≥50 years old with high risk of CV events; not on AHA therapy, or on AHA monotherapy, or combination AHA therapy	156	Canagliflozin 100–300 mg	Placebo
Perkovic et al. (65)	Multicenter	NCT02065791	HbA1c 6.5–12.0%; eGFR 30–90 ml/min/1.73 m^2^; maximum tolerated labeled daily dose of an ACEi or ARB (≥4 weeks); UACR >300 mg/g and ≤ 5,000 mg/g	239	Canagliflozin 100 mg	Placebo
Pratley et al. (66)	Multicenter	NCT02099110	HbA1c 7.5–11.0%; MET ≥1,500 mg/day (≥8 weeks)	54	Ertugliflozin 5 mg/15 mg	Sitagliptin 100 mg
					Ertugliflozin 5 mg/15 mg + Sitagliptin 100 mg	
Qiu et al. (67)	Multicenter	NCT01340664	HbA1c 7.0–10.5%; MET ≥2,000 mg/day or ≥1,500 mg/day (≥8 weeks); FPG <15 mmol/l at week −2; fasting fingerstick glucose 6.1–15 mmol/l on day 1	18	Canagliflozin 50 mg/150 mg BID	Placebo
Ridderstråle et al. (68)	Multicenter	NCT01167881	HbA1c 7.0–10.0%; MET IR ≥1,500 mg/day, maximum tolerated dose, or maximum dose according to the local label (≥3 months)	208	Empaglifozin 25 mg + MET	Glimepiride 1–4 mg + MET
Rodbard et al. (69)	Multicenter	NCT02863328	HbA1c 7.0–10.5 %; MET ≥1,500 mg/day or maximum tolerated (≥3 months)	57	Empagliflozin 25 mg	Semaglutide 14 mg
Roden et al. (70)	Multicenter	NCT01289990	HbA1c 7.0–11%; diet and exercise, drug-naive or pre-treated with pioglitazoneor with MET or pre-treated with MET or MET plus SU at 12 weeks	77	Empagliflozin 10 mg (Drug Naive)	Placebo (Drug Naive)
					Empagliflozin 25 mg (Drug Naive)	Sitagliptin 100 mg (Drug Naive)
					Empagliflozin 10 mg/25 mg (Pioglitazone)	Placebo (Pioglitazone)
					Empagliflozin 10 mg/25 mg (MET)	Placebo (MET)
					Empagliflozin 10 mg/25 mg (MET + SU)	Placebo (MET + SU)
Rosenstock et al. (75)	Multicenter	NCT00683878	HbA1c 7.0–10.5%; C-peptide ≥1.0 ng/ml	48	Dapagliflozin 5 mg/10 mg + Pioglitazone	Placebo + Pioglitazone
Rosenstock et al. (74)	Multicenter	NCT00749190	MET ≥1,500 mg/day or with one other OAD: HbA1c 6.5–9.0%; MET only: HbA1c 7.0–10%; HbA1c 7.0–10.0% at start of placebo run-in period	13	Empagliflozin 1 mg/5 mg/10 mg/25 mg/50 mg	Placebo
						Sitagliptin 100 mg
Rosenstock et al. (72)	Multicenter	NCT01306214	HbA1c 7.5–10%; diet and exercise; treatment with MDI of INS or with MET; MET ≥1,500 mg/day or maximum tolerated dose	52	Empagliflozin 10 mg/25 mg	Placebo
Rosenstock et al. (73)	Multicenter	NCT01011868	HbA1c 7.0–10%; basal INS or with MET and/or SU	82	Empagliflozin 10 mg/25 mg	Placebo
Rosenstock et al. (71)	Multicenter	NCT01809327	Diet and exercise; not on AHA therapy (≥3 months) fingerstick HbA1c 7–12.5%; HbA1c 7.5–12%; FPG ≤ 16.7 mmol/l; fasting fingerstick glucose ≥6.7 mmol/l	30	Canagliflozin 100 mg/300 mg	MET XR
					Canagliflozin 100 mg/300 mg + MET XR	
Ross et al. (76)	Multicenter	NCT01649297	HbA1c 7.0–10%; diet and exercise; MET ≥1,500 mg/day (≥3 months)	17	Empagliflozin 12.5 mg BID/25 mg QD	Placebo
					Empagliflozin 5 mg BID/10 mg QD	
Schernthaner et al. (77)	Multicenter	NCT01137812	HbA1c 7–10.5%; MET ≥2,000 mg/day or ≥1,500 mg/day and SU; FPG <16.7 mmol/l	52	Canagliflozin 300 mg	Sitagliptin 100 mg
Scott et al. (78)	Multicenter	NCT02532855	HbA1c 7.0–9.5%; eGFR 60–90 ml/min/1.73 m^2^; MET (≥1,500 mg/day) or with a SU for ≥8 weeks; fasting fingerstick glucose: 6.1–14.4 mmol/l	26	Dapagliflozin 10 mg	Sitagliptin 100 mg
Seino et al. (79)	Japan	NA	HbA1c 6.9–10.5%, FPG ≥126 mg/dl at weeks −6 or −2; maximum change in body weight of 3.0% between weeks −6 and −2; diet therapy ≥6 weeks	12	Luseogliflozin 1 mg/2.5 mg/5 mg/10 mg	Placebo
Singh et al. (80)	UK	NCT02397421	NYHA functional class I-III HF with prior echocardiographic evidence of LVSD; furosemide ≤ 80 mg daily or equivalent loop diuretic; eGFR ≥45 ml/min/1.73 m^2^; stable HF symptoms with therapy and no hospitalized for HF (≥3 months)	52	Dapagliflozin 10 mg	Placebo
Sone et al. (82)	Japan	NCT02589639	diet and exercise; INS with or without 1 OAD (≥3 months); C-peptide >0.5 ng/ml; INS alone: HbA1c 7.5–10.0%; INS with 1 OAD: HbA1c 7.0–9.5%, placebo run-in period HbA1c 7.5–10.0%	53	Empagliflozin 10 mg/25 mg	Placebo
Stenlof et al. (83)	Multicenter	NCT01081834	Main Study: HbA1c 7–10%, FPG <15 mmol/l; High Glycemic Cohort Sub-study: HbA1c 10–12%, FPG ≤ 19.44 mmol/l	52	Main Study: Canagliflozin 100 mg/300 mg	Main Study: Placebo/Sitagliptin 100 mg
					High Glycemic Sub-study: Canagliflozin 100 mg/300 mg	
Strojek et al. (84)	Multicenter	NCT00680745	HbA1c 7.0–10%; SU monotherapy dose at least half the maximal recommended dose (≥8 weeks)	48	Dapagliflozin 2.5 mg/5 mg/10 mg + Glimepiride	Placebo + Glimepiride
Søfteland et al. (81)	Multicenter	NCT01734785	HbA1c 8.0–10.5%; diet and exercise; MET IR ≥1,500 mg/day, maximum tolerated dose, or maximum dose according to the local label (≥3 months)	25	Empagliflozin 10 mg/25 mg	Placebo
Townsend et al. (85)	Multicenter	NCT01939496	HbA1c 7.0–10%; use 1–3 anti-hyperglycemic agents (no INS); seated office SBP: 130–160 mmHg, seated office DBP ≥70 mmHg; use 1–3 anti-hypertensive agents (no loop diuretics) ≥5 weeks	10	Canagliflozin 100 mg/300 mg	Placebo
Wilding et al. (86)	Multicenter	NCT01106625	HbA1c 7.0–10.5%; MET and SU; FPG <15 mmol/l	52	Canagliflozin 100 mg/300 mg	Placebo
Wilding et al. (87)	Multicenter	NCT00673231	HbA1c 7.5–10.5%; INS ≥30 units/day (≥8 weeks) or with up to 2 OADs; MET ≥1,500 mg/day or maximum tolerated dose and other OADs on at least half the daily maximum dose; diet and exercise	104	Dapagliflozin 2.5 mg/5 mg/10 mg	Placebo
Yale et al. (88)	Multicenter	NCT01064414	HbA1c 7.0–10.5%; eGFR 30–50 ml/min/1.73 m^2^; not on AHA therapy or AHA monotherapy or combination therapy; CKD 3, have generally stable renal function	52	Canagliflozin 100 mg/300 mg	Placebo
Yang et al. (89)	Multicenter	NCT02096705	HbA1c 7.5–11.0% during screening/enrolment; HbA1c 7.5–10.5% 14 days prior to randomization; injectable INS ≥20 IU (≥8 weeks)	28	Dapagliflozin 10 mg	Placebo

*NA, not available; AGI, α-glucosidase inhibitor; DPP-4i, dipeptidyl-peptidase-4 inhibitor; SU, sulphonylurea; TZD, thiazolidinedione; SGLT-2i, sodium-glucose co-transporter 2 inhibitor; MET, metformin; INS, insulin; CKD, chronic kidney disease; AHA, anti-hyperglycemic agent; OHA, oral hypoglycemic agent; OAD, oral anti-diabetic drug; eGFR, estimated glomerular filtration rate; FPG, fasting plasma glucose; QD, once daily; BID, twice daily; XR, extended release; IR, immediate release; BP, blood pressure; SBP, systolic blood pressure; DBP, diastolic blood pressure; ACEi, angiotensin-converting enzyme inhibitor; ARB, angiotensin receptor blocker; UACR, urine albumin to creatinine ratio; Scr, serum creatinine; MDI, multiple daily injection; NYHA, New York Heart Association; HF, heart failure; CV, cardiovascular; CVD, cardiovascular disease; LV, left ventricular; LVSD, left ventricular systolic dysfunction*.

### Risk of Bias Assessment

In most trials, the sequence generation and allocation concealment were low risk of bias, while only one research was unclear for allocation concealment. Five trials had a high risk of bias in the blind method. All the included trials possess low risk of bias for incomplete outcome data. Regarding selective outcome reporting, 11 studies had low risk of bias, while the rest were each unclear risk. Finally, all studies were judged as unclear risk for free of other bias (Supplementary Table 1).

### SGLT-2i vs. Control

The control analysis combined placebo and other hypoglycemic drug trials, and 77 RCTs with 88,973 participants indicated that SGLT-2i had no increase in malignancy overall risk compared to non-SGLT-2i (RR = 1.05, *P* = 0.20). The statistical analysis of SGLT-2i for different types of drugs demonstrated statistical significance in seven studies related to ertugliflozin, which could increase malignant tumor risk in T2D patients (RR = 1.80, 95% CI = 1.02–3.17, *P* = 0.04). For other drugs, including bexagliflozin (RR = 1.25, *P* = 0.69), dapagliflozin (RR = 0.98, *P* = 0.71), empagliflozin (RR = 1.13, *P* = 0.13), canagliflozin (RR = 1.06, *P* = 0.45), tofogliflozin (RR = 1.23, *P* = 0.65) had no significant association with malignant tumors occurrence. Compared with non-SGLT-2i, SGLT-2i was also not linked to tumor incidence in hematological malignancy, digestive system malignancy, malignant breast tumor, malignant skin tumor, malignant tumor of urinary system, malignant tumor of respiratory system, gynecologic malignant tumor, malignant brain tumor, and thyroid malignancy. However, in various SGLT-2i types, dapagliflozin may reduce the incidence of respiratory malignancies, but the difference was not statistically significant (RR = 0.75, 95% CI = 0.54–1.05, *P* = 0.09), while other SGLT-2i types had no impact on the incidence of particular types of tumors. Regarding drug use duration, no significant difference existed in malignant tumor risk between short-, medium-, and long-term drug use (Table 2).

**Table 2:** The incidence of malignant tumors between SGLT-2i and control.

**SGLT-2i vs. control**	**No. of studies**	**Participants**	**RR**	**95% CI**	***p***	**Heterogeneity (*I*^**2**^) (%)**
All	77	88,973	1.05	0.97–1.14	0.20	0
**Type of malignant tumor**						
Hematological malignancy	20	50,817	1.16	0.85–1.60	0.35	0
Ertugliflozin	4	2,093	3.50	0.73–16.83	0.12	0
Dapagliflozin	4	19,464	1.27	0.80–2.00	0.31	0
Empagliflozin	5	11,964	1.29	0.64–2.63	0.48	0
Canagliflozin	5	16,670	0.67	0.35–1.31	0.24	3
Digestive system malignancy	43	68,586	1.05	0.90–1.23	0.54	0
Ertugliflozin	5	4,049	1.83	0.68–4.96	0.23	0
Dapagliflozin	10	22,041	0.94	0.74–1.19	0.59	0
Empagliflozin	12	18,517	1.31	0.92–1.87	0.13	0
Canagliflozin	11	22,476	0.99	0.73–1.33	0.93	0
Breast malignant tumor	31	59,991	1.11	0.85–1.46	0.45	0
Ertugliflozin	3	2,673	1.17	0.38–3.64	0.78	0
Dapagliflozin	11	20,948	1.10	0.73–1.67	0.64	0
Canagliflozin	8	19,705	1.52	0.89–2.60	0.13	0
Skin malignant tumor	23	56,961	1.08	0.87–1.33	0.49	0
Ertugliflozin	3	2,787	1.13	0.41–3.11	0.81	0
Dapagliflozin	11	23,031	1.06	0.77–1.44	0.74	0
Empagliflozin	6	15,171	0.96	0.68–1.36	0.82	0
Canagliflozin	3	15,972	1.71	0.89–3.31	0.11	0
Malignant tumor of urinary system	41	66,633	1.08	0.92–1.27	0.33	0
Ertugliflozin	4	2,661	1.39	0.44–4.38	0.57	0
Dapagliflozin	13	23,721	1.07	0.85–1.33	0.57	0
Empagliflozin	13	18,943	1.00	0.71–1.40	0.99	0
Canagliflozin	9	20,836	1.17	0.85–1.61	0.35	0
Malignant tumor of the respiratory system	26	57,358	0.86	0.69–1.07	0.17	0
Dapagliflozin	6	20,890	0.75	0.54–1.05	0.09	0
Empagliflozin	9	16,390	1.07	0.70–1.64	0.76	0
Canagliflozin	8	19,370	0.82	0.54–1.23	0.33	0
Gynecologic malignant tumor	15	51,139	0.79	0.54–1.16	0.24	0
Dapagliflozin	3	18,068	0.72	0.40–1.32	0.29	0
Empagliflozin	5	12,210	1.42	0.59–3.43	0.44	15
Canagliflozin	6	20,556	0.63	0.33–1.19	0.16	0
Brain malignant tumor	5	29,269	1.10	0.47–2.59	0.83	0
Thyroid malignancy	10	46,115	1.17	0.63–2.20	0.62	0
Canagliflozin	5	16,919	1.89	0.60–5.91	0.27	0
**Types of SGLT-2i**						
Ertugliflozin	7	6,084	1.80	1.02–3.17	*0.04*	0
Bexagliflozin	4	1,274	1.25	0.42–3.73	0.69	0
Dapagliflozin	25	28,994	0.98	0.88–1.10	0.71	0
Empagliflozin	18	24,933	1.13	0.96–1.32	0.13	0
Canagliflozin	18	26,618	1.06	0.91–1.24	0.45	0
Tofogliflozin	3	814	1.23	0.50–3.00	0.65	0
**Follow-up time**						
<52 weeks	30	11,610	1.19	0.80–1.76	0.40	0
52–104 weeks	31	23,804	0.94	0.73–1.21	0.63	0
≥104 weeks	16	53,559	1.06	0.97–1.15	0.18	1

*The values in italics represent statistical differences in the results (i.e., P < 0.05)*.

### SGLT-2i vs. Other Hypoglycemic Drugs

Compared with other hypoglycemic drugs, 25 studies were involved, with 19,703 participants. Among them, 9,917 were SGLT-2i participants, with a total of 100 malignant tumors, and 9,786 were other hypoglycemic drugs, with a total of 94 malignant tumors. Compared with other hypoglycemic drugs, SGLT-2i had no overall risk increase of a malignant tumor (RR = 1.01, *P* = 0.95). Nevertheless, based on SGLT-2i types, empagliflozin (7 studies) was associated with reduced malignant tumor risk (RR = 0.67, 95% CI = 0.45–0.98, *P* = 0.04), while dapagliflozin (6 studies) can upsurge malignant tumor risk (RR = 2.71, 95% CI = 1.46–6.43, *P* = 0.02). Ertugliflozin (RR = 1.74, *P* = 0.12) and canagliflozin (RR = 0.79, *P* = 0.45) were not significantly associated with the malignant tumor risk. By analyzing particular malignant tumor types, empagliflozin data may propose a possible reduction in urinary malignancy risk (RR = 0.48, 95% CI = 0.21–1.10, *P* = 0.08), and dapagliflozin may augment digestive system malignancy occurrence risk (RR = 3.98, 95% CI = 0.85–18.69, *P* = 0.08), but the above differences have no statistical significance. However, compared with other hypoglycemic agents, SGLT-2i did not correlate with malignant tumors incidence in the rest of body. Additionally, follow-up time (short, medium, and long term) had no significant effect on carcinogenic rate of SGLT-2i and had no impact on reducing or increasing malignancy risk compared with different types of active drugs (Table 3).

**Table 3:** The incidence of malignant tumors between SGLT-2i and other hypoglycemic drugs.

**SGLT-2i vs. other hypoglycemic drugs**	**No. of studies**	**Participants**	**RR**	**95% CI**	***p***	**Heterogeneity (*I*^**2**^) (%)**
All	25	19,703	1.01	0.77–1.31	0.95	0
**Type of malignant tumor**						
Hematological malignancy	5	3,321	1.76	0.51–6.01	0.37	0
Digestive system malignancy	16	13,423	1.36	0.82–2.27	0.23	0
Ertugliflozin	3	2,885	1.45	0.43–4.82	0.55	0
Dapagliflozin	4	2,225	3.98	0.85–18.69	0.08	0
Empagliflozin	5	3,824	0.78	0.36–1.73	0.55	7
Breast malignant tumor	12	10,137	1.01	0.54–1.90	0.98	0
Empagliflozin	4	3,138	0.81	0.27–2.44	0.71	0
Canagliflozin	3	3,077	1.10	0.29–4.22	0.89	0
Skin malignant tumor	9	7,572	1.12	0.58–2.18	0.73	0
Dapagliflozin	3	2,052	1.80	0.38–8.45	0.45	0
Empagliflozin	4	3,354	0.89	0.35–2.32	0.82	0
Malignant tumor of urinary system	14	11,616	1.04	0.60–1.81	0.88	0
Empagliflozin	5	4,106	0.48	0.21–1.10	0.08	0
Canagliflozin	4	4,021	1.86	0.55–6.26	0.32	0
Malignant tumor of the respiratory system	8	7,012	0.73	0.33–1.62	0.44	0
Empagliflozin	4	3,305	0.51	0.18–1.48	0.22	0
Gynecologic malignant tumor	8	7,098	0.56	0.26–1.23	0.15	0
Empagliflozin	3	2,532	0.50	0.15–1.71	0.27	2
Canagliflozin	3	3,636	0.41	0.12–1.42	0.16	0
**Types of SGLT-2i**						
Ertugliflozin	3	3,194	1.74	0.86–3.53	0.12	0
Dapagliflozin	6	3,254	2.71	1.14–6.43	*0.02*	0
Empagliflozin	7	5,660	0.67	0.45–0.98	*0.04*	7
Canagliflozin	7	6,871	0.79	0.44–1.45	0.45	0
**Follow-up time**						
<52 weeks	6	2,827	1.72	0.60–4.91	0.31	0
52–104 weeks	12	9,202	0.80	0.53–1.20	0.28	6
≥104 weeks	7	7,674	1.12	0.78–1.63	0.54	0
**Types of hypoglycemic drugs in the control group**						
GLP-1RA	3	2,068	0.71	0.28–1.81	0.48	27
DPP-4i	11	7,106	1.13	0.66–1.92	0.66	0
Sulfonylurea	6	7,496	1.11	0.76–1.62	0.59	0
Metformin	5	2,693	0.55	0.26–1.17	0.12	0

*The values in italics represent statistical differences in the results (i.e., P < 0.05)*.

### SGLT-2i vs. Placebo

A total of 60 SGLT-2i and placebo-controlled studies were included, having 70,600 participants: 36,094 in SGLT-2i group, with 1,150 malignant tumors, and 34,506 in placebo group, with 1,079 malignant tumors. Compared with placebo, SGLT-2i had no overall risk increase of malignancies (RR = 1.05, *P* = 0.20). However, according to different SGLT-2i types, empagliflozin (15 studies) was significantly linked to increased risk of malignancies (RR = 1.25, 95% CI = 1.05–1.49, *P* = 0.01), and the rest of ertugliflozin (*RR* = 1.91, *P* = 0.18), bexagliflozin (RR = 1.08, *P* = 0.90), dapagliflozin (RR = 0.96, *P* = 0.46), canagliflozin (RR = 1.08, *P* = 0.35), and tofogliflozin (RR = 0.76, *P* = 0.70) were not significantly associated with increased malignancies risk. By analyzing specific types of malignancies, SGLT-2i population and each type did not correlate with the incidence of hematological malignancy, malignant skin tumor, malignant tumor of urinary system, malignant tumor of respiratory system, gynecologic malignant tumor, malignant brain tumor, and thyroid malignancy compared with placebo.

Compared with placebo, SGLT-2i overall was not associated with breast cancer incidence (RR = 1.15, *P* = 0.36). Canagliflozin potentially increased breast cancer risk, but the difference was not statistically significant (RR = 1.64, 95% CI = 0.93–2.90, *P* = 0.09). Besides, SGLT-2i population revealed no statistically significant difference in the incidence of digestive system malignancies (RR = 1.01, *P* = 0.92), but empagliflozin (8 studies) was associated with increased risk of digestive system malignancies (RR = 1.48, 95% CI = 0.99–2.21, *P* = 0.05). The data revealed that the follow-up time had no significant impact on malignancy incidence of SGLT-2i (Table 4).

**Table 4:** The incidence of malignant tumors between SGLT-2i and placebo.

**SGLT-2i vs. placebo**	**No. of studies**	**Participants**	**RR**	**95% CI**	***p***	**Heterogeneity (*I*^**2**^) (%)**
All	60	70,600	1.05	0.97–1.14	0.20	0
**Type of malignant tumor**						
Hematological malignancy	16	47,496	1.13	0.81–1.57	0.47	0
Dapagliflozin	4	19,464	1.27	0.80–2.00	0.31	0
Empagliflozin	4	10,277	1.34	0.63–2.87	0.45	0
Canagliflozin	5	16,670	0.67	0.35–1.31	0.24	3
Digestive system malignancy	30	56,264	1.01	0.85–1.19	0.92	0
Dapagliflozin	7	19,816	0.89	0.70–1.14	0.35	0
Empagliflozin	8	14,693	1.48	0.99–2.21	*0.05*	0
Canagliflozin	10	19,812	0.96	0.71–1.30	0.80	0
Breast malignant tumor	23	50,633	1.15	0.85–1.55	0.36	0
Dapagliflozin	9	19,730	1.06	0.69–1.62	0.79	0
Empagliflozin	6	13,187	0.78	0.40–1.54	0.48	0
Canagliflozin	7	17,407	1.64	0.93–2.90	0.09	0
Skin malignant tumor	16	49,389	1.07	0.86–1.34	0.54	0
Dapagliflozin	8	20,979	1.03	0.75–1.42	0.86	0
Empagliflozin	4	11,817	0.97	0.67–1.40	0.88	0
Canagliflozin	3	15,972	1.71	0.89–3.31	0.11	0
Malignant tumor of urinary system	29	55,017	1.09	0.92–1.28	0.33	0
Dapagliflozin	11	22,282	1.05	0.84–1.31	0.70	0
Empagliflozin	10	14,837	1.17	0.80–1.70	0.43	0
Canagliflozin	5	16,815	1.13	0.80–1.58	0.49	9
Malignant tumor of the respiratory system	19	50,346	0.87	0.69–1.09	0.22	0
Dapagliflozin	5	20,076	0.76	0.55–1.06	0.11	0
Empagliflozin	6	13,085	1.23	0.77–1.98	0.39	0
Canagliflozin	6	16,782	0.79	0.52–1.21	0.28	0
Gynecologic malignant tumor	8	44,041	0.88	0.57–1.37	0.57	0
Canagliflozin	4	16,920	0.75	0.35–1.58	0.45	0
Brain malignant tumor	4	27,724	1.23	0.50–3.05	0.65	0
Thyroid malignancy	8	43,740	1.45	0.73–2.90	0.29	0
Canagliflozin	5	16,919	1.89	0.60–5.91	0.27	0
**Types of SGLT-2i**						
Ertugliflozin	4	2,890	1.91	0.74–4.91	0.18	0
Bexagliflozin	3	890	1.08	0.33–3.54	0.90	0
Dapagliflozin	20	25,740	0.96	0.86–1.07	0.46	0
Empagliflozin	15	19,273	1.25	1.05–1.49	*0.01*	0
Canagliflozin	14	21,077	1.08	0.92–1.26	0.35	0
Tofogliflozin	2	474	0.76	0.19–3.02	0.70	0
**Follow-up time**						
<52 weeks	28	10,113	1.07	0.71–1.61	0.74	0
52–104 weeks	22	14,602	1.03	0.75–1.42	0.85	0
≥104 weeks	10	45,885	1.06	0.97–1.15	0.21	14

## Discussion

SGLT-2i possesses good benefits in lowering blood glucose, but some safety problems may result in urogenital infection, bone fractures, ketoacidosis, etc. (90), so its clinical use requires to be considered comprehensively. Epidemiological studies have manifested a link between T2D and cancer, and one of the reasons may be hyperglycemia itself (91). While SGLT-2i may affect malignant tumors occurrence by lowering blood glucose, its comprehensive impact is still uncertain. Tang et al. analyzed 46 RCTs from 24 to 160 weeks and stated that empagliflozin might correlate with increased risk of bladder cancer (11). Nevertheless, the data involved in this analysis were challenged, and the corrected data showcased that empagliflozin might not be linked to bladder cancer (92). Beyond that, Tang et al. also concluded that canagliflozin might have a protective effect on gastrointestinal cancer (11). A meta-analysis of 27 trials displayed that SGLT-2i were not statistically associated with any cancer type (93). The abovementioned studies may be due to the low incidence of malignant tumors, small statistical sample size and short follow-up time, making it challenging to get clear and unified results.

This meta-analysis showed neither a significant association between SGLT-2i and the overall risk of malignancy in T2D patients nor with medication duration, consistent with the results of previous analysis (11, 93, 94). In different types of SGLT-2i analyses, we found that ertugliflozin significantly increased overall malignancy incidence, had no statistically significant difference compared to other hypoglycemic drugs or placebo alone, and had no great risk of a specific malignant tumor. The above could be due to lack of test sample size. A pooled analysis of 7 RCTs concluded that ertugliflozin had no significant difference in malignancies incidence compared with placebo or other active hypoglycemic agents (95).

Moreover, we observed that dapagliflozin might reduce the risk of respiratory system malignancies compared with the control group, but without statistical significance. Sodium-glucose cotransporter 2 (SGLT2) expression increased at the lung premalignancy and early-stage lung adenocarcinoma (96), and dapagliflozin may be able to reduce cancer cells proliferation by inhibiting glucose transport. Villani et al. also found that canagliflozin could prevent lung cancer cells' proliferation by precluding respiration supported by mitochondrial complex-I (97). It is worth noting that our data also indicated that compared with other hypoglycemic drugs, dapagliflozin could increase the overall risk of malignant tumors, and the most likely one was the digestive system malignancy, but without statistically significant difference. According to preceding studies, some active antidiabetic drugs can impede tumors. For example, a meta-analysis by Dicembrini et al. indicates that DPP-4i may have a potential inhibitory effect on colorectal cancer (98). A meta-analysis of 21 studies showed that metformin might be beneficial for survival in patients with pancreatic cancer and diabetes (99). Wu et al. found that metformin had no effect on the overall esophageal cancer risk, but it may reduce esophageal cancer risk in T2D Asian patients (100). Du et al.'s research reported in diabetics of colorectal cancer patients that taking metformin can improve overall survival and cancer specific survival, especially the overall survival of patients with stage II and III (101). A study has indicated that GLP-1RA may not be associated with increased risk of pancreatic cancer (102). In conclusion, other hypoglycemic drugs may be connected with inhibiting digestive system malignancies, making dapagliflozin seemed to be linked to increased incidence of digestive system malignancies. Notably, research has shown that dapagliflozin may have a potential inhibition impact on colon cancer cells expressing SGLT2 but without UDP glucuronosyltransferase family 1 member A9 (UGT1A9) (103). Besides, Okada et al. suggested that dapagliflozin may inhibit tumor growth by inhibiting glucose entry into cancer cells and producing cytotoxic effects in non-metabolized dapagliflozin (104). Canagliflozin may directly reduce liver cancer growth by inhibiting glycolysis and angiogenic activity (105). Tang et al.'s meta-analysis of 35 trials displayed no significant association between SGLT-2i and pancreatic cancer incidence (106). Since our research records the digestive system malignant tumor sample size too small (includes only 4 RCTs, dapagliflozin group 6 cases, and other active hypoglycemic drugs group 0 cases), difference did not reach statistical significance. Accordingly, it is uncertain whether the dapagliflozin increase of digestive system malignancy arising from the overall risk is high.

Simultaneously, our data revealed that compared with other hypoglycemic drugs, empagliflozin could reduce the overall risk of malignant tumors and may reduce the incidence of urinary system malignant tumors, but the latter without statistically significant difference. As previous studies reported, in prostate cancer, SGLT2 is actively expressed and actively participates in glucose uptake, so SGLT-2i may inhibit tumor growth by reducing glucose uptake and disrupting glycolysis (107). Kuang et al. stated that dapagliflozin could induce apoptosis of renal carcinoma cells (108). Data analysis from 20 empagliflozin and placebo-controlled trials revealed no significant association between empagliflozin and the incidence of bladder and renal malignancies (109). An observational, prospective follow-up study has reported that empagliflozin can enhance anti-inflammatory and antioxidant effects in T2D patients, including cardiovascular advantages (110). Anti-inflammatory and antioxidant impacts may also be linked to reduced incidence of malignancies. However, due to the limited number of included RCTs and the difference was not statistically significant, it cannot be confirmed that the overall risk reduction of malignant tumors is ascribed to urinary malignancies reduction. Furthermore, compared with placebo, we stated that empagliflozin significantly increased malignant tumor risk, mainly those of the digestive system. Nevertheless, one summarizes 15 randomized phase I-III trials plus four extension studies of empagliflozin and a placebo-controlled study indicating that empagliflozin safety data had no linkage to T2D patients with malignant tumor (111). The conclusion is still out on whether empagliflozin increases the risk of malignancies.

Our study showcased that canagliflozin might potentially increase breast cancer risk compared with placebo, but no statistically significant difference existed. Earlier data submitted to the Food and Drug Administration (FDA) suggested that dapagliflozin might upsurge breast cancer risk, but subsequent studies have suggested that it may be increased risk due to early cancer diagnosis rather than actual increase in incidence (112). Studies have shown that breast cancer incidence in canagliflozin intervention groups was similar to that in non-canagliflozin groups, and both were lower (113). A large population cohort study with a median of 2.6 years of follow-up showed that SGLT-2i utilization was not associated with increased overall breast cancer risk than DPP-4i (114). Interestingly, a study has implied that canagliflozin holds anti-proliferation effect on breast cancer cells by increasing phosphorylation of adenosine monophosphate activated protein kinase (AMPK) and reducing the phosphorylation of 70 kilodalton (kDa) ribosomal protein S6 kinase 1, thereby blocking cell cycle and inducing apoptosis (115). Besides, a study has manifested that ipragliflozin can inhibit the proliferation of breast cancer cells (116).

The strength of our study lies in the large study scale and sample size. However, our limitations are also obvious. Few studies we referred primarily aimed at assessing the risk of malignancy, and the incidence of malignant tumors closely related to age, diabetes, gender and many other factors. However, due to limited data availability, we can not adjust these parameters. In summary, it is not sufficient to discuss or conclude the relationship between SGLT-2i and malignancy risk in this meta-analysis. More RCTs related to long-term use of SGLT-2i are required in the future to provide more evidence for the safety of such drugs in long-term use.

## Conclusion

In summary, current data from RCTs had no significant association between SGLT-2i and overall malignant tumor risk. Our evidence proposes that ertugliflozin may increase the overall risk of malignancy. Compared with active hypoglycemic agents, dapagliflozin may increase the overall risk of malignant tumor, while empagliflozin may reduce its risk. But compared with placebo, empagliflozin may increase the overall malignancy risk, mainly in the digestive system. However, the follow-up time of the RCTs analyzed in our study were relatively short, and the data of various factors were incomplete, which could insufficient to account for the long-term effects of SGLT-2i on malignant tumors, and more data are required for comprehensive analysis.

## Data Availability Statement

The original contributions presented in the study are included in the article/Supplementary Material, further inquiries can be directed to the corresponding author/s.

## Author Contributions

FX designed the research process. NS and YShi searched the database for corresponding articles. JX and YSi extracted useful information from the articles above. TY and MZ used statistical software for analysis. NS and XL drafted the meta-analysis. DN polished this article. All authors had read and approved the manuscript and ensured that this was the case.

## Conflict of Interest

The authors declare that the research was conducted in the absence of any commercial or financial relationships that could be construed as a potential conflict of interest.

## Abbreviations

SGLT-2i: sodium-glucose cotransporter 2 inhibitor
RCT: randomized controlled trial
RR: risk ratio
CI: confidence interval
T2DM: type 2 diabetes mellitus
T2D: type 2 diabetes
GLP-1RA: glucagon-like peptide-1 receptor agonist
DPP-4i: dipeptidyl-peptidase-4 inhibitor
SGLT2: sodium-glucose cotransporter 2
UGT1A9: UDP glucuronosyltransferase family 1 member A9
AMPK: adenosine monophosphate activated protein kinase
kDa: kilodalton.
