# Supplementary material for: SGLT-2i and Risk of Malignancy in Type 2 Diabetes: A Meta-Analysis of Randomized Controlled Trials
Source: Front Public Health. 2021 Jun 7;9:668368. doi: 10.3389/fpubh.2021.668368 (PMC8215266; doi:10.3389/fpubh.2021.668368)
Supplement: Supplementary file 1 [file Table_1.docx]

**Supplementary Table 1.** Quality assessment of studies included.

| Author, year | Sequence generation | Allocation concealment | Blinding | Incomplete outcome data | Selective outcome reporting | Free of other bias |
| --- | --- | --- | --- | --- | --- | --- |
| Allegretti, 2019 | low risk | low risk | low risk | low risk | unclear risk | unclear risk |
| Araki, 2015 | low risk | low risk | low risk | low risk | unclear risk | unclear risk |
| Aronson, 2018 | low risk | low risk | low risk | low risk | unclear risk | unclear risk |
| Bailey, 2013 | low risk | low risk | low risk | low risk | unclear risk | unclear risk |
| Bailey, 2015 | low risk | low risk | low risk | low risk | unclear risk | unclear risk |
| Barnett, 2014 | low risk | low risk | low risk | low risk | unclear risk | unclear risk |
| Bolinder, 2014 | low risk | low risk | low risk | low risk | unclear risk | unclear risk |
| Brown, 2020 | low risk | low risk | low risk | low risk | unclear risk | unclear risk |
| Böhm, 2020 | low risk | low risk | low risk | low risk | unclear risk | unclear risk |
| Cahn, 2020 | low risk | low risk | low risk | low risk | unclear risk | unclear risk |
| Cefalu, 2015 | low risk | low risk | low risk | low risk | low risk | unclear risk |
| Dagogo-Jack, 2018 | low risk | low risk | low risk | low risk | low risk | unclear risk |
| Ferdinand, 2019 | low risk | low risk | low risk | low risk | low risk | unclear risk |
| Ferrannini, 2013 | low risk | low risk | high risk | low risk | unclear risk | unclear risk |
| Fioretto, 2018 | low risk | low risk | low risk | low risk | unclear risk | unclear risk |
| Forst, 2014 | low risk | low risk | low risk | low risk | low risk | unclear risk |
| Fuchigami, 2020 | low risk | low risk | high risk | low risk | unclear risk | unclear risk |
| Grunberger, 2018 | low risk | low risk | low risk | low risk | unclear risk | unclear risk |
| Gallo, 2019 | low risk | low risk | low risk | low risk | unclear risk | unclear risk |
| Hadjadj, 2016 | low risk | low risk | low risk | low risk | unclear risk | unclear risk |
| Halvorsen, 2019 | low risk | low risk | low risk | low risk | unclear risk | unclear risk |
| Halvorsen, 2019 | low risk | low risk | low risk | low risk | low risk | unclear risk |
| Halvorsen, 2020 | low risk | low risk | low risk | low risk | unclear risk | unclear risk |
| Haneda, 2016 | low risk | unclear risk | high risk | low risk | unclear risk | unclear risk |
| Henry, 2012 | low risk | low risk | low risk | low risk | unclear risk | unclear risk |
| Hollander, 2019 | low risk | low risk | low risk | low risk | unclear risk | unclear risk |
| Ikeda, 2015 | low risk | low risk | low risk | low risk | unclear risk | unclear risk |
| Inagaki, 2013 | low risk | low risk | low risk | low risk | unclear risk | unclear risk |
| Inagaki, 2014 | low risk | low risk | low risk | low risk | unclear risk | unclear risk |
| Jabbour, 2014 | low risk | low risk | low risk | low risk | low risk | unclear risk |
| Jabbour, 2020 | low risk | low risk | low risk | low risk | low risk | unclear risk |
| Januzzi, 2017 | low risk | low risk | low risk | low risk | unclear risk | unclear risk |
| Ji, 2014 | low risk | low risk | low risk | low risk | unclear risk | unclear risk |
| Ji, 2019 | low risk | low risk | low risk | low risk | unclear risk | unclear risk |
| Katakami, 2020 | low risk | low risk | high risk | low risk | unclear risk | unclear risk |
| Kadowaki, 2015 | low risk | low risk | low risk | low risk | unclear risk | unclear risk |
| Kadowaki, 2017 | low risk | low risk | low risk | low risk | unclear risk | unclear risk |
| Kaku, 2013 | low risk | low risk | low risk | low risk | unclear risk | unclear risk |
| Kaku, 2014 | low risk | low risk | low risk | low risk | unclear risk | unclear risk |
| Kawamori, 2018 | low risk | low risk | low risk | low risk | unclear risk | unclear risk |
| Kohan, 2014 | low risk | low risk | low risk | low risk | unclear risk | unclear risk |
| Lavalle-González, 2013 | low risk | low risk | low risk | low risk | unclear risk | unclear risk |
| Leiter, 2014 | low risk | low risk | low risk | low risk | unclear risk | unclear risk |
| Leiter, 2015 | low risk | low risk | low risk | low risk | unclear risk | unclear risk |
| Lewin, 2015 | low risk | low risk | low risk | low risk | unclear risk | unclear risk |
| Lingvay, 2019 | low risk | low risk | low risk | low risk | unclear risk | unclear risk |
| Mathieu, 2016 | low risk | low risk | low risk | low risk | unclear risk | unclear risk |
| Matthaei, 2015 | low risk | low risk | low risk | low risk | unclear risk | unclear risk |
| Müller-Wieland, 2018 | low risk | low risk | low risk | low risk | unclear risk | unclear risk |
| Nauck, 2014 | low risk | low risk | low risk | low risk | unclear risk | unclear risk |
| Oshima, 2020 | low risk | low risk | low risk | low risk | unclear risk | unclear risk |
| Oshima, 2020 | low risk | low risk | low risk | low risk | unclear risk | unclear risk |
| Perkovic, 2019 | low risk | low risk | low risk | low risk | low risk | unclear risk |
| Pratley, 2018 | low risk | low risk | low risk | low risk | low risk | unclear risk |
| Qiu, 2014 | low risk | low risk | low risk | low risk | unclear risk | unclear risk |
| Ridderstråle, 2018 | low risk | low risk | low risk | low risk | unclear risk | unclear risk |
| Rodbard, 2019 | low risk | low risk | high risk | low risk | unclear risk | unclear risk |
| Roden, 2015 | low risk | low risk | low risk | low risk | unclear risk | unclear risk |
| Rosenstock, 2012 | low risk | low risk | low risk | low risk | unclear risk | unclear risk |
| Rosenstock, 2013 | low risk | low risk | low risk | low risk | unclear risk | unclear risk |
| Rosenstock, 2014 | low risk | low risk | low risk | low risk | unclear risk | unclear risk |
| Rosenstock, 2015 | low risk | low risk | low risk | low risk | unclear risk | unclear risk |
| Rosenstock, 2016 | low risk | low risk | low risk | low risk | low risk | unclear risk |
| Ross, 2015 | low risk | low risk | low risk | low risk | unclear risk | unclear risk |
| Seino, 2014 | low risk | low risk | low risk | low risk | unclear risk | unclear risk |
| Schernthaner, 2013 | low risk | low risk | low risk | low risk | unclear risk | unclear risk |
| Scott, 2018 | low risk | low risk | low risk | low risk | low risk | unclear risk |
| Stenlöf, 2014 | low risk | low risk | low risk | low risk | unclear risk | unclear risk |
| Singh, 2020 | low risk | low risk | low risk | low risk | unclear risk | unclear risk |
| Sone, 2020 | low risk | low risk | low risk | low risk | unclear risk | unclear risk |
| Strojek, 2014 | low risk | low risk | low risk | low risk | unclear risk | unclear risk |
| Søfteland, 2017 | low risk | low risk | low risk | low risk | unclear risk | unclear risk |
| Townsend, 2016 | low risk | low risk | low risk | low risk | unclear risk | unclear risk |
| Wilding, 2013 | low risk | low risk | low risk | low risk | unclear risk | unclear risk |
| Wilding, 2014 | low risk | low risk | low risk | low risk | unclear risk | unclear risk |
| Yale, 2014 | low risk | low risk | low risk | low risk | unclear risk | unclear risk |
| Yang, 2018 | low risk | low risk | low risk | low risk | unclear risk | unclear risk |

The RCTs were assessed by the Cochrane Collaboration’s tool. Risk of bias was assessed as “low risk”, “high risk” or “unclear risk”.
